# Naturally occurring mutations associated with resistance to HCV NS5B polymerase and NS3 protease inhibitors in treatment-naïve patients with chronic hepatitis C

**DOI:** 10.1186/s12985-015-0414-1

**Published:** 2015-11-14

**Authors:** Angela Costantino, Enea Spada, Michele Equestre, Roberto Bruni, Elena Tritarelli, Nicola Coppola, Caterina Sagnelli, Evangelista Sagnelli, Anna Rita Ciccaglione

**Affiliations:** Department of Infectious, Parasitic and Immune-Mediated Diseases, Viral Hepatitis Unit, Istituto Superiore di Sanità, Viale Regina Elena, 299-00161 Rome, Italy; Department of Cell Biology and Neurosciences, Istituto Superiore di Sanità, Rome, Italy; Department of Mental Health and Public Medicine, Section of Infectious diseases, Second University of Naples, Naples, Italy; Department of Experimental Medicine and Surgery F. Magrassi and A. Lanzara, Second University of Naples, Naples, Italy

**Keywords:** Hepatitis C virus, Mutations, Antiviral treatment, Genotype, Italy

## Abstract

**Background:**

The detection of baseline resistance mutations to new direct-acting antivirals (DAAs) in HCV chronically infected treatment-naïve patients could be important for their management and outcome prevision. In this study, we investigated the presence of mutations, which have been previously reported to be associated with resistance to DAAs in HCV polymerase (NS5B) and HCV protease (NS3) regions, in sera of treatment-naïve patients.

**Findings:**

HCV RNA from 152 naïve patients (84 % Italian and 16 % immigrants from various countries) infected with different HCV genotypes (21,1a; 21, 1b; 2, 2a; 60, 2c; 22, 3a; 25, 4d and 1, 4k) was evaluated for sequence analysis. Amplification and sequencing of fragments in the NS5B (nt 8256–8640) and NS3 (nt 3420–3960) regions of HCV genome were carried out for 152 and 28 patients, respectively. The polymorphism C316N/H in NS5B region, associated with resistance to sofosbuvir, was detected in 9 of the 21 (43 %) analysed sequences from genotype 1b-infected patients. Naturally occurring mutations V36L, and M175L in the NS3 protease region were observed in 100 % of patients infected with subtype 2c and 4.

**Conclusion:**

A relevant proportion of treatment naïve genotype 1b infected patients evaluated in this study harboured N316 polymorphism and might poorly respond to sofosbuvir treatment. As sofosbuvir has been approved for treatment of HCV chronic infection in USA and Europe including Italy, pre-treatment testing for N316 polymorphism on genotype 1b naïve patients should be considered for this drug.

## Background

Hepatitis C virus (HCV) is a global health problem. Approximately 3 % of the world population is infected with HCV, the leading cause of liver cirrhosis, hepatic decompensation, development of hepatocellular carcinoma (HCC) and liver transplantations worldwide [1]. HCV is characterized by a high genetic heterogeneity and is classified in 7 genotypes and numerous subtypes [2]. Some subtypes are ubiquitous while others circulate in particular regions [3]. In Italy, the prevalence of HCV is declining. Recent studies carried out among general population in both northern and southern Italy have reported an anti-HCV prevalence ranging from 2.6 to 5.7 [4–6]. However it is a common opinion among researchers that at present in Italy the prevalence of HCV chronic carriers is about 1–1.5 %. The most frequent genotypes circulating in general population are 1b, 2c and, to a less extent, 3a, while the prevalence of genotype 4 has increased in recent years, in southern regions [6–8]. Pegylated-Interferon plus ribavirin (peg-IFN-RBV) has been the standard-of-care treatment for chronic hepatitis C for many years, although it had some disadvantages, such as a suboptimal therapy response rate in some patients’ subsets (genotype 1 and 4 infections, advanced liver fibrosis) and side effects often resulting in poor tolerability and treatment contraindication [9]. Recently, new anti-HCV drugs, termed direct-acting antiviral agents (DAAs) have been developed. DAAs target different regions of HCV genome and can be distinguished in NS3/4A protease inhibitors (PIs), NS5A inhibitors, NS5B nucleoside (NPIs) and non-nucleoside polymerase inhibitors (NNPIs). Boceprevir and Telaprevir, first generation NS3 PIs, were approved in the 2011 in United States by Food and Drug Administration and Europe (FDA) by European Medicines Agency (EMA) for therapy of chronic hepatitis C. These drugs, however, administered in combination with peg-IFN and ribavirin were hampered by a low efficacy against non-1 genotypes, serious adverse reactions and low genetic barrier to resistance. In addition to the first generation PIs, two new NS3 PIs (Simeprevir and Faldaprevir) one NS5A inhibitor (Dataclasvir) and one NS5B NPI (Sofosbuvir) have been recently (FDA, EMA, 2014) approved for treatment of chronic hepatitis C [10]. The most recent DAAs increase response rates, allow shortened and simplified regimens and have a higher genetic barrier to resistance [11]. However, the effectiveness of new DAAs may be affected by the development of drug-resistance mutations. Due to the high sequence diversity, high replication rate and low fidelity of HCV RNA-polymerase, numerous variants, defined as quasispecies, exist *in vivo* in a HCV infected patient [12]. Some of these variants can carry amino-acid substitutions which determine conformation changes of a drug binding site, thus causing resistance during therapy [13, 14]. These drug resistance substitutions, which usually emerge after a few days of DAAs treatment and are responsible for treatment failure (particularly with first generation drugs) [15, 16] or hyporesponsiveness to treatment, can also be found in HCV infected treatment-naïve patients [17–23]. These naturally resistant variants have been reported to occur at variable frequencies and are genotype/subtype dependent. In fact, the frequency of natural resistance mutations to first generation NS3 PIs is lower in genotype 1b compared to genotype 1a patients [19]. Resistance mutations to NS3 PIs in treatment-naïve patients infected with non-1 genotypes have been investigated in several studies, but only one of them detected two principal mutations (V158M for genotype 2c and D168E for genotype 4) in a significant number of patients infected with genotypes 2c and 4 [23]. On the contrary, many substitutions associated with resistance to NS3 PIs have been reported for genotype 1 [24]. The S282T mutation in NS5B polymerase region, identified *in vitro* [25] and *in vivo* in a 2b infected patient who failed therapy during a clinical trial [26, 27], is the only mutation so far surely associated with resistance to sofosbuvir. Indeed, several other NS5B substitutions have also been suggested as responsible for sofosbuvir treatment failure [28]. In particular, a baseline NS5B polymorphism at position 316 has been potentially associated with reduced response rates to sofosbuvir in genotype 1b patients [29]. The aim of this study was to investigate the presence of variants resistant to DAAs in the NS5B polymerase and NS3 serine protease regions by analysing patients with chronic hepatitis C who had not been treated with any DAAs.

## Materials and methods

This study included 152 DAA-naïve patients chronically infected with HCV genotype 1a (*n* = 21), 1b (*n* = 21), 2a (*n* = 2), 2c (*n* = 60), 3a (*n* = 22), 4d (*n* = 25) and 4 k (*n* = 1) [6–8], and analysed for NS5B region (nt 8256–8640) using primers and amplification conditions reported elsewhere [4, 6–8]. For 28 (11, 1b; 11, 2c and 6, 4d) of these 152 patients, HCV-NS3 region (3420–3960) was also amplified by nested PCR using in the first round primers and amplification conditions previously reported [30]. In the second round, new designed primers and a different thermal cycle program were used, while all other amplification conditions (buffer, primers’ concentration, etc.) were the same as those used in the first PCR round (Table 1). An automated DNA sequencer (Beckman Coulter, Inc., Fullerton, CA) was used for sequencing. Nucleotide sequences were assembled using Chromas software, and aligned using ClustalW package.

**Table 1:** Primers used for HCV NS3 protease gene amplification

HCV genotype	I-PCR primers	II-PCR primers	5’-3’ sequence^b^	H77 location
1b	MarsF2^a^ MarsR1^a^	NS31b F^b^ MarsR2^a^	5'- TRCCHGTCTCCGCCCGVAG-3'^c^	3291-3310 4215–4237 3340–3358 4035-4054
2c	MarsF1^a^ MarsR1^a^	NS3 2c F^b^ NS3 2c R^b^	5'- TGGGCCCTGCTGATGGATAC-3'^d^ 5'- GTAGGTCTGGGGCACAGCTG-3'	3291-3310 4215–4237 3376–3395 4010-3991
4d	G4F1^a^ G4R1^a^	NS3 4d F^b^ NS3 4d R^b^	5’-ATGCGCACRCYAYGAAGGG-3’^e^ 5’-GGCACGGCAGGAGGAGTGGA-3’	3369-3388 3997–4018 3388–3407 4000-3981

^a^ Previously published primers (Vallet S [30])

^b^ Primers selected by alignment of sequences from Los Alamos databank

^c^ Amplification conditions in the second PCR for genotype 1b, were: 2’ at 94 °C, and then 30 cycles at 94 °C for 30”, 60 °C for 30” and 72 °C for 45”, plus extension at 72 °C for 7’

^d^ Amplification conditions in the second PCR for genotype 2c, were: 2’at 94 °C, and then 30 cycles at 94 °C for 30”, 62 °C for 30” and 72 °C for 45”, plus extension at 72 °C for 7’

^e^ Amplification conditions in the second PCR for genotype 4, were: 2’at 94 °C, and then 30 cycles at 94 °C for 30”, 60 °C for 40” and 72 °C for 45”, plus extension at 72 °C for 7’

## Results

A total of 152 patients (81 males and 55 females; median age 47, range 20–90 years) were analysed. About 84 % (127/152) of these patients were of Italian origin, while the remaining 25 patients were immigrants from various countries. From sera of these patients were obtained 152 NS5B and 28 NS3 sequences. All aminoacid substitutions in NS5B polymerase and NS3 protease regions potentially associated with resistance to DAAs in HCV- infected treatment-naive patients in this study, are shown in Tables 2 and 3, respectively. The NS5B polymerase fragment of all analysed genotypes showed no mutation known to induce high level of resistance to sofosbuvir *in vitro* (S282T), as well as other mutations recently regarded as responsible for sofosbuvir treatment failure in clinical trials (V321I/A, L320F/C) [22]. Whereas, the polymorphism C316N/H, potentially associated with reduced response rates to sofosbuvir in genotype 1b HCV chronically infected patients [23], was found in 9 of 21 (43 %) analysed 1b sequences (C316N and C316H polymorphisms were detected in 8 and in 1 patients, respectively). No substitutions conferring resistance to both first generation and new NS3 PIs (Simeprevir and Faldaprevir), were observed in the NS3 region of genotype 1b sequences. Instead, the V36L and M175L substitutions, know to induce decreased susceptibility exclusively to first generation PIs in genotype 1 infections, were naturally present in NS3 region of genotype 2c and 4d sequences.

**Table 2:** Aminoacid substitutions in HCV NS5B polymerase region associated with resistance to DAAs^a^ in treatment-naïve patients

R^b^	P^c^	D^d^	M^e^	Drug	G 1a (*n* = 21)	G 1b (*n* = 21)	G 2a (*n* = 2)	G 2c (*n* = 60)	G 3a (*n* = 20)	G 4d (*n* = 25)	G 4 k (*n* = 1)
NS5B	316	C	N,Y,H	Sofosbuvir, Mericitabine	•	^*^C(57)/N(38),H(5)	•	•	•	•	•
NS5B	282	S	T	Sofosbuvir, Mericitabine	•	•	•	•	•	•	•
NS5B	320	I		Sofosbuvir, Mericitabine	•	•	•	•	•	•	•
NS5B	321	V		Sofosbuvir, Mericitabine	•	•	•	•	•	•	•

^a^ direct acting antiviral agents

R^b^, Region; P^c^, Position aa; D^d^, Dominant aa; M^e^, Mutant aa; (Donaldson, [29]; Lam, [25]; Tong, [28])

*Percentage of sequences with substitutions

•The dominant residue is found in 100 % of analyzed sequences for each genotype

**Table 3:** Aminoacid substitutions in HCV NS3 protease region associated with resistance to DAAs^a^ in treatment-naïve patients

R^b^	P^c^	D^d^	M^e^	Drug	G 1b (*n* = 11)	G 2c (*n* = 11)	G 4d (*n* = 6)
NS3	16	C	S	ACH806	*C/R(9)	*A(100)	*T(100)
NS3	36	V	A,M,G,L	Telaprevir, Boceprevir	*V(100)	L(100)^f^	L(100)^f^
NS3	54	T	A,S	Telaprevir, Boceprevir	^•^	^•^	^•^
NS3	155	R	K,T,I,MG,L,S,Q	Telaprevir, Boceprevir	^•^	^•^	^•^
NS3	156	A	S,T,V,I	Telaprevir, Boceprevir	^•^	^•^	^•^
NS3	168	D	Q,A,V,E,T	TMC435,R7227,MK7009, BI201335, BILN2061	^•^	^•^	^•^
NS3	175	M	L	Telaprevir	*M(100)	L(100)^f^	L(100)^f^

^a^ direct acting antiviral agents

R^b^, Region; P^c^,Position aa; D^d^,Dominant aa; M^e^, Mutant aa; (Lopez-Labrador, 2008; Lentz, 2010; Kieffer [15], Halfon [13], Kuntzen [19])

*Percentage of sequences with substitutions

•The dominant residue is found in 100 % of analyzed sequences for each genotype

^f^ Substitution found is associated with resistance in genotype 1b

Finally, several polymorphisms not associated with resistance to DAAs and already reported by others [18] were observed in NS3 region of our analysed-genotypes (Tables 4, 5 and 6).

**Table 4:** Polymorphic sites in the HCV 1b NS3 protease region found in this study

^a^Position	^b^Subtype 1b	^a^Position	^b^Subtype 1b
7	S7/A (6)	114	V114/I (11)
14	L14/V (1)	122	S122/T (1)
30	D30/E (10)	128	S128/Y (1)
48	V48/I (6)	131	P131/T (1)
51	V51/A (1)	132	I132/V/L (5)
56	Y56/F (4)	142	P142/L (1)
61	S61/P/T (5)	150	V150/A (9)
83	V83 /I (1)	170	I170/V (5)
86	P86/Q (11)	178	T178/V (2)
87	A87/S (2)		
94	M94/L (10)		
107	V107/I (1)		

^a^The residues are reported as in the wild-type HCV-1a sequence

^b^The number of patients with mutant HCV strains is indicated in brackets

**Table 5:** Polymorphic sites not associated with resistance to DAAs in the HCV 2c NS3 protease

Position NS3^a^	Subtype 2c^b^
15	D15G/S (9)
33	V33I (3)
35	V35I (1)
134	S134T (4)
146	P146S (1)
177	V177I (1)

^a^The residues are reported as in the wild-type HCV-1a sequence

^b^The number of patients with mutant HCV strains is indicated in brackets

**Table 6:** Polymorphic sites in the HCV-4 NS3 protease not resistance-associated found in this study

^a^Positions	^b^Subtype 4	^a^Positions	^b^Subtype 4
12	G12/R (1)	98	T98/A (1)
13	L13/M (8)	102	A102/S (8)
14	F14/L (8)	105	Y105/F (7)
15	S15/G (7)	114	I114/V (8)
18	V18/I (8)	122	T122/N (1)
24	R24/K (1)	127	L127/I (5)
33	V33/I (4)	133	S133/A (1)
47	A47/S (3)	134	I134/T (8)
48	V48/I (7)	147	M147/L (8)
61	A61/S (8)	150	R150/A (8)
92	R92/K (8)	172	V172/I (1)
95	A95/T (1)	179	M179/A (1)

^a^ The residues are reported as in the wild-type HCV-1a sequence

^b^ The number of patients with mutant HCV strains is indicated in brackets

## Discussion

This study reports the results of the HCV sequencing from 152 treatment-naïve patients chronically infected with different HCV genotypes. In these patients, we evaluated the presence of known drug resistance mutations focusing on NS5B polymerase and NS3 protease regions. Aminoacid substitutions, such as V36L and M175L, associated with HCV-genotype 1 PIs resistance *in vivo *and *in vitro*, were naturally observed in 100 % of our patients infected with genotype 2c and 4d. The impact of this latter finding on therapy is unclear. Indeed, boceprevir and telaprevir showed less efficacy against genotype 2, and almost no efficacy against other non-1 genotypes, including genotype 4. We cannot exclude that the reported low efficacy of boceprevir and telaprevir against genotype 2 and 4 is influenced by the presence of these natural polymorphisms detected by us and others [23]. The mutations in the NS3 region are rare to detect in naïve patients. Unfortunately, for tecnical reason the sequence analysis of the NS3 region in this study was limited to 28 serum samples only. However, these descriptive frequency data could be useful for further studies that analyse this HCV RNA region in the prediction of HCV antiviral therapy outcome. With regard to the presence of pre-treatment sofosbuvir resistance mutations, the S282T variant was not found in any of our patients, regardless of genotype. This is in agreement with literature data reporting that S282T variants are mainly found *in vitro* [25]. It is likely that our negative result, regarding the detection of this mutation, may be also due to the limits of the sequencing method used in this study. Indeed, the Sanger method can potentially misrepresent the totality of viral quasispecies harboured by an HCV infected patient [27]. In fact, in a recent study the S282T variant has been detected by deep sequencing at 0.05 % of viral sequences at baseline in a genotype 2b infected subject who relapsed following 12 weeks of SOF monotherapy [27]. Besides, other S282 variants (S282R/G/C) have been identified in naïve subjects [31].

Recently, novel (treatment-emergent) NS5B mutations (L159F, L320F and V321A) were detected in HCV infected patients who failed sofosbuvir treatment [29]. Importantly, a baseline polymorphism at position 316 has been associated with sofosbuvir treatment failure in genotype 1b infected patients [29]. It has been reported that C316 mutation is positioned closed to the catalytic triad of the NS5B polymerase and changes at this position can prevent binding of sofosbuvir [29]. We have found substitution 316 N/H in 43 % of genotype 1b sequences. The same position, as well as positions 320 and 321, was highly conserved (100 %) in the other analysed genotypes. The identification of baseline resistance mutations to DAAs could be important for patients’ management and outcome prevision. In this regard, the detection of 316 N/H polymorphism in 43 % of our 1b naive infected patients represents the most relevant finding of this study since these patients might poorly respond to sofosbuvir therapy. Indeed, a recent study reported that baseline polymorphisms at position 316 were potentially associated with reduced response rates in HCV genotype 1b subjects [29]. In conclusion, pre-treatment testing for C316N polymorphism on HCV genotype 1b seems to be indicated for naïve patients considered for treatments including sofosbuvir.

## Abbreviations

HCV: Hepatitis C virus
IFN: Interferon
RBV: Ribavirin
DAAs: Direct-acting antiviral agents
PIs: Protease inhibitors
NS5B-NPIs: NS5B nucleoside polymerase inhibitors
NS5B –NNPIs: NS5B non-nucleoside polymerase inhibitors
NS3: Non-structural region 3
NS5B: Non-structural region 5B
